# Development and Validation of a Novel Dual Luciferase Reporter Gene Assay to Quantify Ebola Virus VP24 Inhibition of IFN Signaling

**DOI:** 10.3390/v10020098

**Published:** 2018-02-24

**Authors:** Elisa Fanunza, Aldo Frau, Marco Sgarbanti, Roberto Orsatti, Angela Corona, Enzo Tramontano

**Affiliations:** 1 Department of Life and Environmental Sciences, University of Cagliari, 09124 Cagliari, Italy; elisafanunza@unica.it (E.F.); aldofrau@unica.it (A.F.); angela.corona@unica.it (A.C.); 2 Department of Infectious Diseases, Istituto Superiore di Sanità, 00161 Rome, Italy; marco.sgarbanti@iss.it (M.S.); roberto.orsatti@iss.it (R.O.); 3 Genetics and Biomedical Research Institute, National Research Council, 09042 Monserrato, Italy

**Keywords:** Ebola virus VP24, innate immunity, IFN signaling, IFN inhibition, dual luciferase gene reporter assay, drug development

## Abstract

The interferon (IFN) system is the first line of defense against viral infections. Evasion of IFN signaling by Ebola viral protein 24 (VP24) is a critical event in the pathogenesis of the infection and, hence, VP24 is a potential target for drug development. Since no drugs target VP24, the identification of molecules able to inhibit VP24, restoring and possibly enhancing the IFN response, is a goal of concern. Accordingly, we developed a dual signal firefly and Renilla luciferase cell-based drug screening assay able to quantify IFN-mediated induction of Interferon Stimulated Genes (ISGs) and its inhibition by VP24. Human Embryonic Kidney 293T (HEK293T) cells were transiently transfected with a luciferase reporter gene construct driven by the promoter of ISGs, Interferon-Stimulated Response Element (ISRE). Stimulation of cells with IFN-α activated the IFN cascade leading to the expression of ISRE. Cotransfection of cells with a plasmid expressing VP24 cloned from a virus isolated during the last 2014 outbreak led to the inhibition of ISRE transcription, quantified by a luminescent signal. To adapt this system to test a large number of compounds, we performed it in 96-well plates; optimized the assay analyzing different parameters; and validated the system by calculating the Z′- and Z-factor, which showed values of 0.62 and 0.53 for IFN-α stimulation assay and VP24 inhibition assay, respectively, indicative of robust assay performance.

## 1. Introduction

Ebola virus (EBOV) causes sporadic outbreaks of severe hemorrhagic fever in humans and nonhuman primates with fatality rates as high as 90%. The EBOV outbreak in 2014 is one of the largest viral outbreaks in history and the first in West Africa, causing over 11,000 deaths in Africa and other parts of the world [1]. EBOV is an enveloped negative-sense single-stranded RNA virus that belongs to the family of *Filoviridae*. Although the molecular determinants of EBOV pathogenesis remain still incompletely defined, the ability of the virus to counteract early antiviral responses, particularly the antagonism of the type I interferon (IFN-I) response, is thought to contribute to its high virulence [2,3,4]. The IFN system is the first line of defense against viral infections. It begins with recognition of a pathogen-associated molecular pattern (PAMP) by pattern recognition receptors (PRRs), whose activation leads to the production of IFN-I (IFN-α/β). Binding of IFN-I to the cell surface IFN-I receptor (IFNAR) initiates a signaling cascade that results in the activation and phosphorylation of the Janus kinases, Jak1 and Tyk2, and the Signal Transducer and Activator of Transcriptions 1 and 2 (STAT1 and STAT2). Phosphorylated STAT1 (P-STAT1) either dimerizes or forms a heterotrimeric transcriptional complex with STAT2 and the IFN Regulatory Factor 9 (IRF9), named IFN-Stimulated Gene Factor 3 (ISGF3), and is subsequently transported to the nucleus via karyopherin-α (KPNA) where it regulates the expression of hundreds of IFN-stimulated genes (ISGs) that target specific aspects of the viral life cycle [5]. Among the seven major EBOV gene products, two viral proteins have been shown to suppress host IFN response. Viral protein 35 (VP35) blocks the IFN production cascade by binding double-stranded (ds)RNA and shielding it from recognition by host immune sensors such as the Retinoic acid-Inducible Gene I (RIG-I) and the Melanoma Differentiation-Associated protein 5 (MDA-5) [4,6,7]; in addition, it also interferes with the activation of the IFN regulatory factor 3 (IRF3) [7,8,9,10,11] and prevents RIG-I interaction with the Protein kinase R activator, PACT [12,13]. Viral protein 24 (VP24) inhibits signaling downstream of both IFN-α/β and IFN-γ by sequestering KPNA proteins (α1, α5, and α6) and preventing nuclear transport of activated P-STAT1 [14,15,16]. In addition, VP24 also binds directly to P-STAT1 [17]. EBOV evasion of IFN signaling by VP24 is a critical event in the pathogenesis of the infection. Mutations in VP24 correlated with IFN evasion are responsible for the acquisition of high virulence in animal models [18]. Being a key factor in EBOV virulence, VP24 is a potential target for the development of new drugs. In the absence of approved drugs specific for VP24, the identification of molecules able to inhibit VP24, restoring and possibly enhancing the IFN response, is a goal of concern. We describe here the development of a novel artificial dual cell-based gene reporter screening assay able to quantify IFN induction and its inhibition by VP24. Human Embryonic Kidney 293T (HEK293T) cells transiently express the human Interferon-Stimulated Response Element (ISRE) driving a luciferase reporter gene. Stimulation with human recombinant IFN-α activates the IFN signaling cascade leading to the expression of ISGs genes. This system may be a suitable cellular model to reproduce the effect of VP24 on IFN signaling and to test VP24 inhibitors. The enzymatic activity of firefly luciferase provides a susceptible, stable, and rapid means to quantify transcriptional activity of ISRE. We optimized the assay, evaluating different parameters to achieve an excellent signal. Further, the normalization with a Renilla luciferase control allows us to minimize variability between experiments, providing high reproducibility of our dual drug screening assay.

## 2. Materials and Methods

### 2.1. Cells and Reagents

HEK293T cells (ATCC^®^ CRL-3216™) were grown in Dulbecco’s modified Eagle’s medium (Gibco, Waltham, MA, USA) supplemented with 10% fetal bovine serum (Gibco) and 1% penicillin/streptomycin (Sigma, St. Louis, MO, USA). Cells were incubated at 37 °C in a humidified 5% CO_2_ atmosphere. Plasmid pISRE-luc was kindly provided by Prof. Ian Goodfellow. Plasmid pcDNA3-V5-VP24 was a kind gift of Dr. St Patrick Reid. pcDNA3-NS1 was kindly given by Prof. Stephan Ludwig. pRL-TK was purchased from Promega (Madison, WI, USA). T-Pro P-Fect Transfection Reagent was from T-Pro Biotechnology (Taipei, Taiwan R.O.C.). Human Recombinant IFN-α was purchased from PeproTech (London, UK). Mouse monoclonal anti-FLAG M2 was obtained from Sigma, and rabbit monoclonal anti-GAPDH and HRP-linked anti-rabbit IgG were purchased from Cell Signaling (Danvers, MA, USA). HRP-linked anti-mouse IgG was provided by Invitrogen (Carlsbad, CA, USA). Pierce ECL Western Blotting Substrate was from Thermo Fisher Scientific (Waltham, MA, USA).

### 2.2. Construction of EBOV VP24 Mammalian Expression Plasmid

The EBOV VP24 cDNA was obtained by de novo gene synthesis (GenScript USA Inc. Piscataway, NJ, USA) using the sequence of VP24 from the Zaire Ebolavirus isolate H.sapiens-wt/GIN/2014/Gueckedou-C05, Sierra Leone/Guinea. The FLAG epitope, present in frame at the NH_2_ terminus of VP24 sequence, and the HindIII and EcoRV restriction sites at both ends, used for the subcloning in the pcDNA3.1(+) mammalian expression vector (Invitrogen), were also part of the entire sequence obtained by de novo gene synthesis.

### 2.3. Luciferase Reporter Gene Assay

HEK293T cells were transfected using T-Pro P-Fect Transfection Reagent, according to the manufacturer’s protocol. Cells (1.5 × 10^4^ cells/well) were seeded in 96-well plates 24 h before transfection. Plasmids pISRE-luc, pRL-TK (Promega) were mixed in reduced serum medium Optimem (Gibco) and incubated with transfection reagent for 20 min at room temperature. Transfection complexes were then gently added into individual wells of the 96-well plate. Twenty-four hours after transfection, cells were stimulated with Human Recombinant IFN-α and incubated for 8 h at 37 °C in 5% CO_2_. Cells were harvested with 50 μL of lysis buffer (50 mM Na-2-(*N*-Morpholino)ethanesulfonic acid(MES) (pH 7.8), 50 mM Tris-HCl (pH 7.8), 1 mM dithiothreitol, and 0.2% Triton X-100). To the lysates were added 50 μL of luciferase assay buffer (125 mM Na-MES (pH 7.8), 125 mM Tris-HCl (pH 7.8), 25 mM magnesium acetate, and 2.5 mg/mL ATP). Immediately after addition of 50 μL of 1 mM d-luciferin, the firefly luminescence was measured in a Victor3 luminometer (Perkin Elmer, Waltham, MA, USA). An equal volume, 50 μL, of coelenterazine assay buffer (125 mM Na-MES (pH 7.8), 125 mM Tris-HCl (pH 7.8), 25 mM magnesium acetate, 5 mM KH_2_PO_4_, 10 μM coelenterazine) was added and the bioluminescence was read. The relative light units (RLU) were normalized as the fold induction over unstimulated controls. Each assay was performed in triplicate.

### 2.4. EBOV VP24 Inhibition Assay

EBOV VP24 inhibition assay was performed in 96-well plates. The construct pISRE-luc was cotransfected with pcDNA3.1-FLAG-VP24 or pcDNA3-V5-VP24. Empty vector pcDNA3.1(+) was used as the negative control and Influenza type A Virus (IAV) nonstructural protein 1 (NS1) expression plasmid as the positive control of inhibition. pRL-TK (Promega) was used as the internal control for transfection efficiency. Concentrations of plasmids are indicated in the figures’ legends. Luciferase activity was expressed as the percentage of EBOV VP24 transfected samples values normalized to the empty vector (indicated as 100%).

### 2.5. Immunoblot Analysis

To detect EBOV VP24 expression levels, HEK293T cells were seeded in 6-well plates (4 × 10^5^ cell/mL) and transfected with pISRE-luc and increasing concentrations of EBOV FLAG-VP24 plasmid. Twenty-four hours post-transfection, the medium was replaced with IFN-α. Eight hours post-IFN addition, cells were harvested and lysed in radioimmunoprecipitation (RIPA) buffer (50 mM Tris-HCl (pH 8), 150 mM NaCl, 1% Triton X-100, 0.5% sodium deoxycholate, 0.1% sodium dodecyl sulfate, 1 mM phenylmethylsulfonyl fluoride, 1× Roche complete protease inhibitor cocktail, 1 mM sodium orthovanadate). Total cell extracts were analyzed by sodium dodecyl sulfate-polyacrylamide gel electrophoresis (SDS-PAGE) and then transferred to a polyvinylidene difluoride (PVDF) membrane by standard methods. Membranes were blocked with 3% nonfat dry milk in Tris Buffered Saline (50 mM Tris-HCl, 0.138 M NaCl, 2.7 mM KCl, pH 8.0) and probed with mouse monoclonal anti-FLAG M2 and rabbit monoclonal anti-GAPDH. Secondary antibodies were HRP-linked anti-mouse IgG and HRP-linked anti-rabbit IgG. Detection was performed using Pierce ECL Western Blotting Substrate and the Chemidoc MP Imaging System (Bio-Rad, Hercules, CA, USA).

### 2.6. Data Analysis

To determine the concentration of IFN-α that provides the maximal activation of the JAK-STAT pathway and to calculate the concentration of EBOV VP24 plasmid required to inhibit 50% of IFN-α stimulation (half maximal inhibitory concentration, IC_50_), we used a three-parameter concentration–response curve as described previously [19] using the log agonist concentration versus response, variable slope algorithm in GraphPad Prism software (GraphPad Prism 6, La Jolla, CA, USA), where:

(1) $$Y\;=\mathrm{Bottom}\;+\;\left(\mathrm{Top}-\mathrm{Bottom}\right)/\left(1+10\text{ˆ}\left({\mathrm{LogIC}}_{50}\right)\right)$$

The IC_50_ value was calculated based on three independent experiments performed in triplicate. The Z- and Z′-factor values were calculated from the following equations developed by Zhang et al. [20]:

(2) $$Z=1-\frac{\left(3\times {\mathrm{SD}}_{s}+3\times {\mathrm{SD}}_{c}\right)}{\left|M_{s}-M_{c}\right|}$$

(3) $$Z^{\prime }=1-\frac{\left(3\times {\;\mathrm{SD}}_{c+}+3\times {\mathrm{SD}}_{c-}\right)}{\left|M_{c+}-M_{c-}\right|}$$

where SD and M are the standard deviation and mean, s and c represent sample and control, and c+ and c− represent positive and negative controls, respectively. For ISRE activation assay we calculated the Z′-factor, considering c+ and c− as the stimulated control and the not-stimulated control, respectively. For VP24 assay we considered the Z-factor, where s are samples cotransfected with pISRE-luc and VP24 and c are cells cotransfected with pISRE-luc and empty vector. All experiments were repeated at least three times. Quantitative data were expressed as the mean ± SD.

## 3. Results

### 3.1. Establishment of a Miniaturized Cell-Based Assay for Evaluating VP24 Inhibition of JAK/STAT Cascade Activation

**Figure 1:**
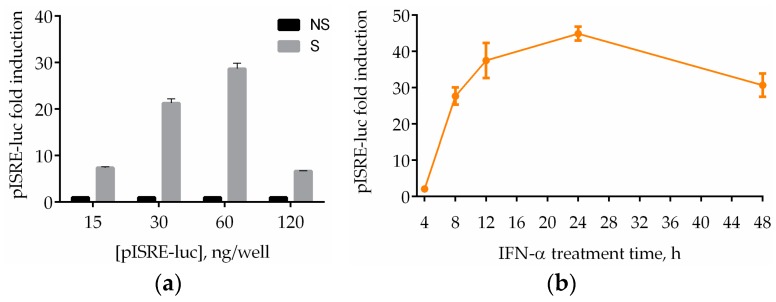
Miniaturized luciferase reporter gene assay in 96-well plates. (**a**) Human embryonic kidney 293T (HEK293T) cells were transfected with 15, 30, 60, and 240 ng of pISRE-luc per well. Twenty-four hours after transfection, cells were stimulated (S) or not (NS) with interferon-α (IFN-α) for IFN signaling pathway activation. After 8 h, cells were lysed and luciferase activity was measured; (**b**) HEK293T cells were transfected with 60 ng/well of pISRE-luc. Twenty-four hours after transfection, cells were stimulated with IFN-α. After 4, 8, 12, 24, 48 h, cells were harvested and luciferase activity was measured. Results are shown as pISRE-luc fold induction of stimulated cells over the not-stimulated control. Error bars indicate the mean ± SD.

To monitor the activation status of IFN signaling, we transfected HEK293T cells with pISRE-luc, a reporter plasmid that is widely used to evaluate the transcription effect of activated JAK/STAT pathway by type I IFNs [21,22]. Treatment with human recombinant IFN-α of the transiently transfected HEK293T cells activates the IFN signaling pathway leading to a luminescent signal. To use this assay to test a considerable number of compounds, we performed it in 96-well plates. For initial assay development, we analyzed the concentration of luciferase plasmid to use for transfection. Hence, HEK293T cells were transfected with various amounts of plasmid per well. Results showed that the optimal concentration for the reporter vector was 60 ng/well (Figure 1a). To assess the optimal time of stimulation, we performed a time-course analysis. HEK293T cells exhibited an IFN-α treatment time for maximal signal between 12 and 24 h (Figure 1b). We chose the 8 h time point to allow strong stimulation, with luciferase activity increased by 27-fold compared with in cells not treated with IFN.

**Figure 2:**
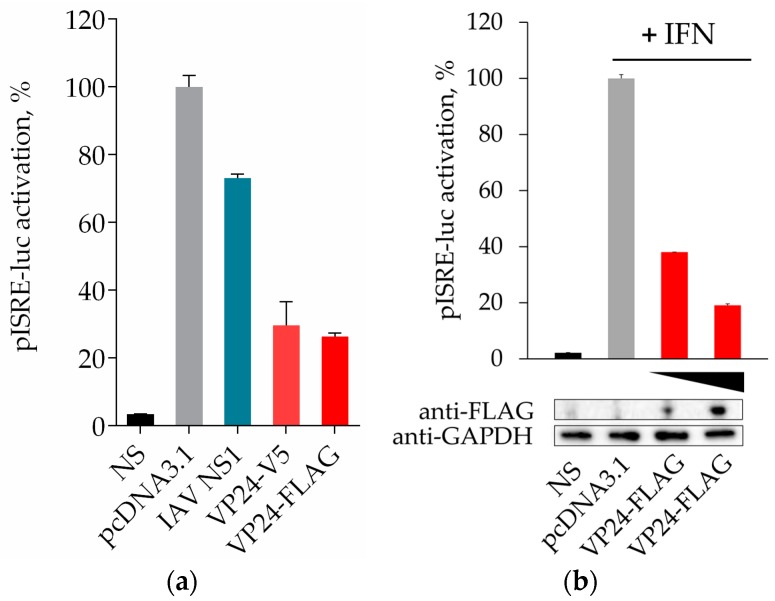
Ebola virus (EBOV) VP24 inhibition assay. (**a**) HEK293T cells were transfected with pISRE-luc and 60 ng/well of empty vector pcDNA3.1(+), or expression plasmid for EBOV FLAG- or V5-tagged VP24 and IAV NS1. Twenty-four hours after transfection, cells were stimulated with 10 ng/mL of IFN-α or left untreated (NS). After 8 h, cells were lysed and luciferase activity was measured. (**b**) Expression of EBOV VP24 at protein level. HEK293T were seeded in a 6-well plate. After 24 h, cells were transfected with pISRE-luc and pcDNA3.1 (1 μg/well) and 0.25 and 1 μg/well of EBOV VP24-FLAG. The day after, cells were incubated or not with IFN-α for 8 h and subsequently lysed and examined for luciferase activity and protein expression by Western blotting. Results are shown as percentage of pISRE-luc activation in VP24 transfected cells over empty vector transfected controls. Each experiment was done in triplicate. Error bars indicate the mean ± SD.

The ability of EBOV VP24 to inhibit the IFN signaling pathway has been demonstrated [14,15,16]. Given the contribution of VP24 to the EBOV virulence [18,23], the protein could be a very important target for drug development. For this reason, we wanted to evaluate its inhibitory effect in our system, to provide a suitable model for screening a large quantity of potential inhibitors. HEK293T were cotransfected with pISRE-luc reporter and empty vector pcDNA3.1(+), or expression plasmid for the FLAG- or V5-tagged VP24. We also cotransfected cells with the expression plasmid for IAV NS1, a previously demonstrated inhibitor of IFN signaling, as a positive control [24]. Cells were stimulated with IFN-α for 8 h. A 30-fold induction in ISRE activity was observed in IFN-α treated samples transfected with pISRE-luc with or without empty vector. This activation was inhibited in cells expressing either FLAG- or V5-tagged VP24 protein and in cells expressing NS1, but with different percentages (Figure 2a). In order to demonstrate that the inhibitory effect of ISRE transcription was due effectively to VP24 expression at the protein level, we performed a Western blot of the samples transfected with EBOV VP24-FLAG or empty vector treated or not with IFN-α and, in parallel, assayed the luciferase activity. Results clearly showed that higher amounts of expressed VP24 corresponded to higher inhibition of the pathway (Figure 2b), demonstrating that the dose-dependent inhibition of the luciferase activity was due to the dose-dependent expression of VP24.

### 3.2. Optimization of Stimulation Conditions and Evaluation of VP24 IC_50_

**Figure 3:**
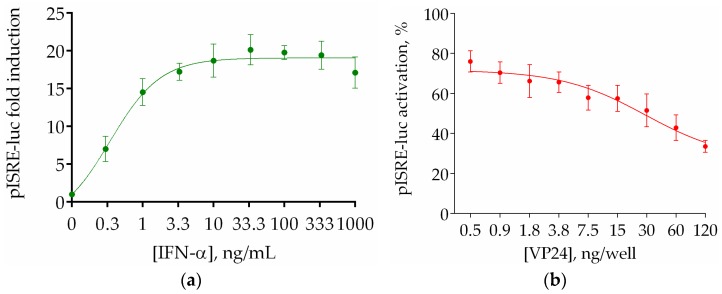
(**a**) HEK293T cells were transfected with pISRE-luc (60 ng/well), RL-TK (10 ng/well), and pcDNA3.1 (30 ng/well). Twenty-four hours after transfection, cells were stimulated with different concentrations of IFN-α between 0.3 and 1000 ng/mL or left unstimulated. The medium was changed after 8 h, and luciferase activity was measured. Results are shown as pISRE-luc fold induction of stimulated cells over not stimulated control. Firefly luciferase activity is normalized to the Renilla luciferase internal control. Error bars indicate the mean ± SD; (**b**) Regression curve of VP24 inhibition was obtained transfecting HEK293T cells with reporter vector at the fixed concentration of 60 ng/well, RL-TK (10 ng/well), and EBOV VP24-FLAG or empty vector in concentrations between 0.5 and 120 ng/well. Results are shown as percentage of pISRE-luc activation in VP24 transfected cells over empty vector transfected controls. Firefly luciferase activity is normalized to the Renilla luciferase internal control. Error bars indicate the mean ± SD.

Normalization with an internal control is crucial to reduce variability between experiments and to develop a drug screening assay. In order to assess the optimal concentration of IFN-α to use for quantifying the dose-dependent effect of VP24 in our inhibition assay, we performed a curve of IFN-α using Renilla luciferase as internal control for transfection. As shown in Figure 3a, IFN-α activated the ISRE reporter gene expression concentration dependently. For inhibition assay, the concentration of agonist required to achieve a “maximum” signal response corresponds to the 80% of stimulation, accordingly with acceptance criteria for assay validation [25]. Hence, we used the concentration at the initial inflexion of the IFN curve (1 ng/mL) to perform the VP24 dose–response assay. Cotransfection of HEK293T with concentrations between 0.5 and 120 ng/well were utilized to create the dose-dependence inhibition curve (Figure 3b) and the 50% inhibitory concentration (IC_50_) was calculated (30 ng/well). Thus, we chose this concentration for further drug screening assay development.

### 3.3. Luciferase Assay Signal Stability

**Figure 4:**
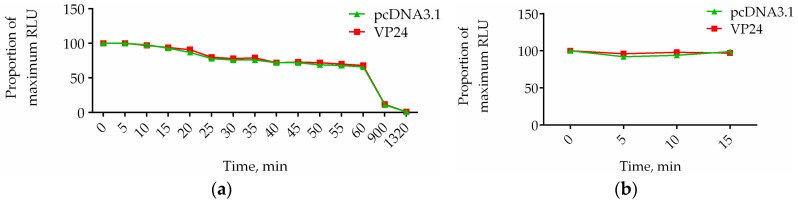
Time-dependent decay of luciferase activity. HEK293T cells cotransfected with pISRE-luc and empty vector or pISRE-luc and VP24 were lysed. (**a**) After addition of d-luciferin, luciferase signal was read over 1320 min. The relative decay is reported by plotting relative light units (RLU) as a proportion of the RLU at t_0_ (100%); (**b**) Addition of d-luciferin was performed at 0, 5, 10, 15 min after incubation with luciferase assay buffer. The luciferase signal is reported by plotting RLU as a proportion of the RLU at t = 0 min after addition of luciferase buffer (100%).

The stability of the luciferase signal is a relevant factor supporting drug screening applications. The time-dependent decay of RLU generated from our firefly luciferase activity was explored. After adding d-luciferin, the luminescent signal generated from cells transfected with ISRE alone and with ISRE and VP24 was recorded every 5 min. As it is shown by the luminescence decay curve (Figure 4a) the maximum level of bioluminescence was reached 5 min after substrate addition, followed by slow decay over 1 h. Because reading a 96-well plate requires the readout signal to be stable for about 20 min (10 s/well), to minimize timing effects we decided to read lines two by two, avoiding the problem of decay of the signal. The time needed by the luminometer to read two lines is 4.30 min, time in which the signal is perfectly stable. We wanted to verify that the addition of luciferase substrate at 0, 5, 10, and 15 min after the incubation with luciferase assay buffer for Lines 1 and 2, Lines 3 and 4, Lines 5 and 6, and Lines 7 and 8, respectively, does not affect the intensity of signal obtained. In Figure 4b we show that even if harvesting and luciferase assay buffer are present for longer times in the other lines compared to the first two, the intensity of the signal does not change. The luminescence signal is expressed as percentage over the value (taken to be 100%) at time t = 0 min (t_0_) that represents the lines 1 and 2 where d-luciferin is immediately added.

### 3.4. Assay Validation

To achieve optimization, coefficients of variation (CV) were calculated for the not-stimulated controls (Min signal), for cells cotransfected with pISRE-luc and pcDNA3.1 (Max signal) or VP24 (IC_50_) (Mid signal) after stimulation with the maximum signal response concentration of IFN-α (1 ng/mL). Representative results are provided in Table 1. CVs for each signal are less than 20%, indicating achieved acceptance criteria [25].

In order to assess the plate uniformity and to evaluate the signal variability between different days, we performed inter-plate and inter-day tests. Plate-to-plate or day-to-day variation in 50% activity needs to be assessed during validation of a drug screening assay [25]. Thus, we performed the tests evaluating as the midpoint the signal obtained transfecting cells with the IC_50_ of VP24 plasmid (30 ng/well). In Table 2 are indicated the percentages of ISRE expression in the presence of VP24 (IC_50_). The fold shift is not higher than 2 for both the inter-day and inter-plate tests, confirming acceptance criteria for validation.

**Figure 5:**
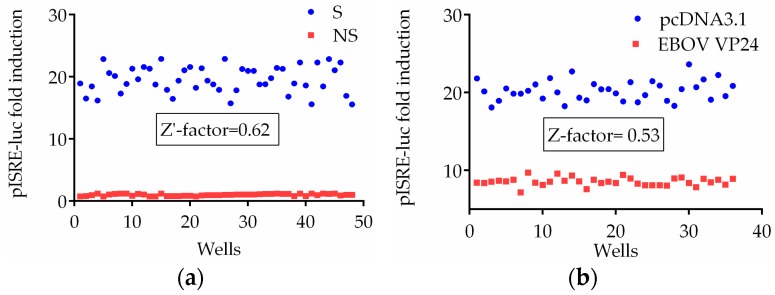
Z′- and Z-factor determination for IFN-α stimulation and VP24 inhibition assays. Firefly luciferase activity is normalized to the Renilla luciferase internal control. Results are expressed as pISRE-luc fold induction over the unstimulated control. The Z′- and Z-factor were calculated as described in methods. (**a**) HEK293T cells were seeded in a 96-well plate and transfected with pISRE-luc (60 ng/well) and pcDNA3.1 (30 ng/well). Twenty-four hours post-transfection, 48 wells (S) were incubated with 1 ng/mL of IFN-α and 48 wells were left untreated (NS). (**b**) HEK293T cells seeded in 48 wells were transfected with pISRE-luc (60 ng/well) and pcDNA3.1 (30 ng/well), and those in the remaining 48 wells were transfected with pISRE-luc (60 ng/well) and expression plasmid for VP24 (30 ng/well). Thirty-five wells for each sample were stimulated with 1 ng/mL of IFN-α and 12 were not stimulated.

Using the optimized reaction conditions previously calculated, the assay procedures were validated in a Z-factor experiment. The Z-factor is a measure of assay quality that takes into account the standard deviation, the dynamic range between positive and negative controls, and the signal variation amongst replicates. A scale of 0–1 is used, with values greater than or equal to 0.5 indicative of an excellent assay. For ISRE activation assay we calculated the Z′-factor using HEK293T cells untreated or treated with IFN-α as negative and positive controls, respectively. For inhibition assay, the Z-factor was measured considering the difference in signal between cells cotransfected with pISRE-luc and empty vector or pISRE-luc and VP24 expression plasmid. As shown in Figure 5, the Z′- and Z-value for each assay plate were 0.62 for stimulation assay and 0.53 for inhibition assay, indicating the robustness of the system.

### 3.5. Designing the Drug Screening Assay

**Figure 6:**
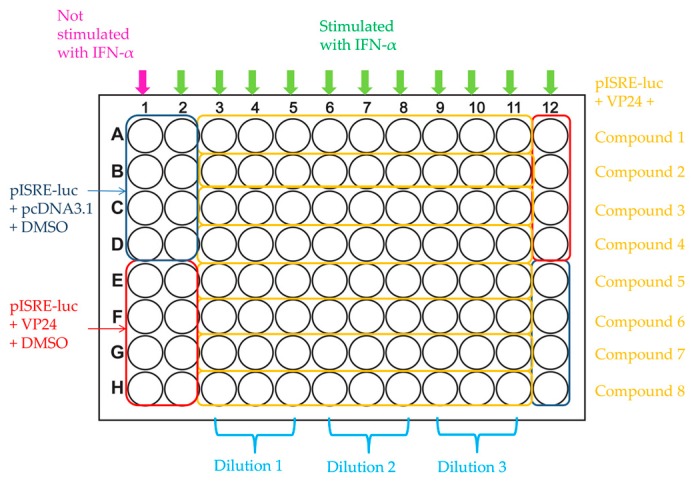
Representative scheme of 96-well plate for drug screening.

For drug screening assay, HEK293T cells (1.5 × 10^4^ cells/well) are cotransfected with pISRE-luc and empty vector or VP24 expression plasmid (IC_50_). At 24 h post transfection, the cells are mock treated or treated with IFN-α in the presence of compound at three different concentrations or dimethyl sulfoxide (DMSO). A concentration of IFN-α (0.3 ng/mL) in the linear range of the IFN-α dose–response curve is used. At 8 h post-treatment, cells are harvested, and firefly and renilla luciferase activities are measured. Positive controls of inhibition are cells cotransfected with pISRE-luc and VP24 in presence of DMSO, while negative controls of inhibition are cells cotransfected with pISRE-luc and empty vector in presence of DMSO. Luciferase activity is calculated as folds induction of stimulated versus not-stimulated controls as well as percentage of pISRE-luc activation in VP24 transfected cells over empty vector transfected controls. A plate map illustrating the layout of the screening plate, including well locations of compounds and positive and negative controls, is represented in Figure 6.

### 3.6. IFN-α Partially Reverts VP24 Inhibition of JAK/STAT Cascade

**Figure 7:**
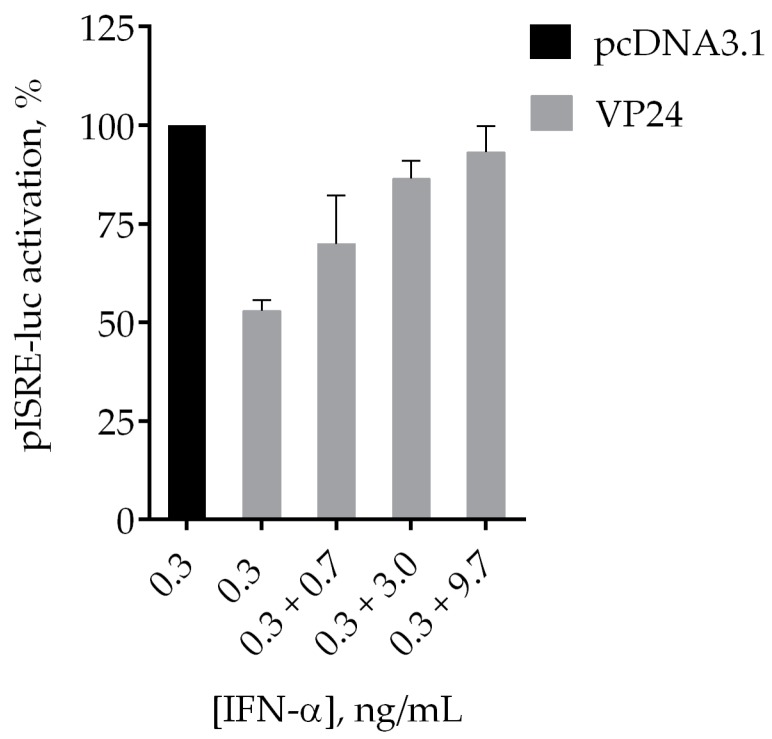
Effect of IFN-α on VP24 inhibition of IFN signaling. HEK293T cells were cotransfected with pISRE-luc (60 ng/well), RL-TK (10 ng/well), and pcDNA3.1 or VP24 expression plasmid (30 ng/well). Twenty-four hours after transfection, cells were stimulated with 0.3 ng/mL of IFN-α. Then, increasing concentrations of IFN-α were added. After 8 h of stimulation, the medium was changed and luciferase activity was measured. Results are shown as percentage of ISRE expression in VP24 transfected cells over empty vector transfected control. Firefly luciferase activity is normalized to the Renilla luciferase internal control. Error bars indicate the mean ± SD.

The lack of inhibitors for VP24 inhibition of the JAK/STAT cascade does not allow a positive control of restoration of the JAK/STAT cascade activation in presence of VP24. However, to investigate the suitability of the assay for drug screening, we evaluated the reversion of the effect of VP24 on pISRE-luc activation mediated by IFN-α. HEK293T cells were cotransfected with pISRE-luc and empty vector or VP24 expression plasmid at 30 ng/mL (IC_50_). In addition to the standard amount of IFN-α (0.3 ng/mL), we added increasing concentrations of IFN-α (0.7, 3, 9.7 ng/mL). As shown in Figure 7, IFN-α is able to partially revert the ISRE expression in presence of VP24, demonstrating its inhibitory effect against the viral protein.

## 4. Discussion

Ebola virus has evolved multiple strategies to antagonize the IFN-α/β responses in target cells such as macrophages, monocytes, and dendritic cells, resulting in total impairment of the innate immune system. An important contribution to EBOV virulence is given by the protein VP35 through its suppression of IFN production [6,7,8,9,12], but a crucial role is exerted by another viral protein, VP24, that disables cells for viral replication and propagation by interfering with IFN signaling [14,15,16,17,18,26,27]. Since it is clear that the early control of viremia is one of the keys for survival, blocking EBOV VP24 action on the cellular immune response is an important subject for investigation and, hence, a validated pharmacological target.

Much work has been done in the last years to elucidate the function of EBOV VP24 in IFN antagonism. These efforts have been conducted employing different methods. Immunofluorescence analysis with multiple epitope tags, such as phospho-specific antibodies for P-STAT1 or STAT1 fused to green fluorescent protein (GFP), was used to demonstrate the ability of VP24 to inhibit IFN-induced nuclear localization and tyrosine phosphorylation of human STAT1. An equivalent effect was confirmed after EBOV infection of cells [14,15,16]. Co-immuonoprecipitation studies revealed that VP24 selectively interacts with KPNA (α1, α5, and α6), competing with P-STAT1 for the same binding [14,15], and in vitro ELISA assays were used to show that purified EBOV VP24 could also bind directly to purified truncated STAT1 [17]. Subsequently, characterization of the binding of EBOV VP24 to KPNA was performed by isothermal titration calorimetry (ITC), in vitro pull down assay with recombinant expressed proteins, and computational analysis [28]. In addition, X-ray crystallization analysis allowed the determination of the complex structure [28]. Affinity tagging coupled to label-free quantitative mass spectrometry was used to identify potential interacting partners of VP24 and confirmed the binding with KPNA [29]. Reverse genetics was used to identify the mutations correlated with the ability to evade type I IFN responsible for the acquisition of the high virulence of the adapted Ebolavirus Mayinga strain in mice [18]. Among the variety of techniques, the use of luciferase reporter assays to measure viral antagonist activity against the IFN system has long been established to be robust and biologically relevant [14,15,16].

Our group has previously developed a luciferase assay gene reporter able to quantify the inhibition of the IFN system by EBOV VP35 that is used for testing compounds against this protein [30]. Since with such a system we could study the inhibition of the first cascade of IFN production, the RIG-I pathway, we decided to develop a new miniaturized luciferase assay that could be used to study the second cascade of the IFN system, the JAK-STAT pathway. We report here its use to measure the EBOV VP24 inhibition of the IFN signaling in HEK293T cells, to be used as a screening system to identifying EBOV VP24 inhibitors as well as to dissect the interaction between EBOV VP24 and its cellular partners.

Experiments were carried out in 96-well plates, always at least in triplicate, allowing good statistical evaluation of the results and showing good reproducibility. In addition, the use of 96-well plates allows us to perform the entire assay in only one plate, avoiding a more complex experimental approach. Luciferase assays done in 6-, 12-, 24-, and 48-well plates imply that cell lysates are moved from a big plate to another with more passages and risk losing material. One advantage of this assay is that the luminescence signal derives effectively from all cells in the well and that the technique is absolutely faster than traditional methods.

Various parameters of the microtransfection method were optimized: amount of plasmids, amount of IFN-α and stimulation time. Measurements of luciferase activity performed at various times yielded optimal results between 8 and 24 h post transfection. Thus, a shorter 8 h incubation was chosen. In literature, luciferase assay performed to evaluate EBOV VP24 inhibition requires at least 16 h of stimulation to measure the luminescence signal [14,15,23,31,32]. The present method allows data to be obtained more rapidly. Conventional transfection assays also require the use of large quantities of plasmids and reagents [14,15,23], while in the present system, as little as 60 ng/well for plasmid reporter can be used, compared to 0.5–1 μg/well in a 6-well plate [14,23]. Hence, the present method appears to be extremely sensitive and allows considerable economy of all reagents. Moreover, the use of homemade reagents for reading luciferase activity is a convenient and ultra-low-cost system compared to the use of highly expensive commercial kits. 

Our assay provides a convenient tool for large-scale screening studies to identify selective EBOV VP24 inhibitors as well as compounds subverting the VP24 block of the IFN cascade. In fact, all acceptance criteria for drug screening validation, including the Z’-factor analysis, were satisfied. In addition, the expression of Renilla luciferase offers an internal control value with which the firefly luciferase reporter gene is normalized, providing a decrease in variability and an increase in consistency. We confirmed the feasibility of the present assay to be used for a rapid and quantitative evaluation of anti-EBOV VP24 agents. Indeed, the experiment with the increasing concentrations of IFN-α suggested that it is possible to override the inhibitory effect of VP24 and partially restore the IFN signaling in our system.

In conclusion, the development of a new dual cell-based assay able to quantify the inhibition of IFN signaling by EBOV VP24 could have important implications for future studies since it represents a suitable model for screening compounds against a viral target for which no drugs are currently approved. The enzymatic activity of luciferase and its normalization with the Renilla internal control allows us to achieve robust assay performance as the Z- and Z′-factor calculations demonstrate. Overall, the present method offers multiple advantages such as 100- to 1000-fold reduction in cells, plasmids, and reagents required, shorter time assay, reduced costs, and rapid detection of molecules that are anti-EBOV VP24.

## Figures and Tables

**Table 1:** Well-to-well reproducibility of viral protein 24 (VP24) inhibition assay.

Signal	Coefficients of Variation, CV (%)
Min	6.1
Mid	4.6
Max	3.3

**Table 2:** Inter-plate and Inter-day tests for VP24 inhibition assay.

Inter-Plate Test	pISRE-luc Activation (%)	Inter-Day Test	pISRE-luc Activation (%)
Plate 1	52.4	Plate 1	49.3
Plate 2	50.8	Plate 2	49.0
